# In Search of Complementary Extraction Methods for Comprehensive Coverage of the *Escherichia coli* Metabolome

**DOI:** 10.3390/metabo13091010

**Published:** 2023-09-14

**Authors:** Henry Gould, William Cheung, James D. Finnigan, José Muñoz-Muñoz, Simon J. Charnock, Gary W. Black

**Affiliations:** 1 Hub for Biotechnology in the Built Environment, Department of Applied Sciences, Faculty of Health and Life Sciences, Northumbria University, Newcastle upon Tyne NE1 8ST, UK; william.cheung@northumbria.ac.uk (W.C.); jose.munoz@northumbria.ac.uk (J.M.-M.); gary.black@northumbria.ac.uk (G.W.B.); 2 Prozomix Limited, West End Industrial Estate, Haltwhistle NE49 9HA, UK; james.finnigan@prozomix.com (J.D.F.); simon.charnock@prozomix.com (S.J.C.)

**Keywords:** *Escherichia coli*, metabolomics, extraction methods, mass spectrometry

## Abstract

*Escherichia coli* is an invaluable research tool for many fields of biology, in particular for the production of recombinant enzymes. However, the activity of many such recombinant enzymes cannot be determined using standard biochemical assays, as often, the relevant substrates are not known, or the products produced are not detectable. Today, the biochemical footprints of such unknown enzyme activities can be revealed via the analysis of the metabolomes of the recombinant *E. coli* clones in which they are expressed, using sensitive technologies such as mass spectrometry. However, before any metabolites can be identified, it is necessary to achieve as high a coverage of the potential metabolites present within *E. coli* as possible. We have therefore analyzed a wide range of different extraction methods against the cell free extracts of various recombinant *E. coli* clones. The results were analyzed to determine the minimum number of extractions that achieved high recovery and coverage of metabolites. Two methods were selected for further analysis due to their ability to produce not only high numbers of ions, but also wide mass coverage and a high degree of complementarity. One extraction method uses acetonitrile and water, in a 4:1 ratio, which is then dried down and reconstituted in the chromatography running buffer prior to injection onto the chromatography column, and the other extraction method uses a combination of methanol, water and chloroform, in a 3:1:1 ratio, which is injected directly onto the chromatography column. These two extraction methods were shown to be complementary to each other, as regards the respective metabolites extracted, and to cover a large range of metabolites.

## 1. Introduction

*Escherichia coli* currently has >2600 identified metabolites that make up its metabolome, as well as many more that have yet to be identified [1]. It is widely used as a tool for recombinant protein expression. The expression of such recombinant enzymes in *E. coli* will change its metabolome [2]. Consequently, metabolomics can be used as a tool for discovering the function of enzymes of unknown specificity. However, there is a considerable challenge when trying to identify metabolic changes in *E. coli*, as in order to be certain of metabolic changes, it is necessary to have a complete picture of all metabolites present. Historically, much of the focus, with regard to metabolite extraction, has been to use one extraction method and try to maximize the efficiency of the extraction solvent. Indeed, some studies suggest that it is possible to achieve this effectively using a single extraction method to reduce resource consumption [3]. For example, many metabolomic studies on bacteria use a 1:3 chloroform to methanol mixture or a 4:1 ratio of methanol to water [4]. Some studies have also suggested that using acetonitrile as one of the major organic components is more effective in extracting polar metabolites than use of either methanol or water [5]. These methods are used for much of metabolomics, as they tend to yield a higher number of polar metabolites than non-polar, as nearly all products of primary metabolism are polar. Additionally, there are numerous methods now that focus on extracting non-polar molecules and lipids from samples [6]. These biphasic extractions take advantage of the partitioning of molecules of different polarities between an aqueous phase and a chloroform-rich phase (for example, the Folch method [7]), although there has been little research that looks at the aqueous-rich phase of these biphasic methods. Finally, it is necessary to mention that some harsher extraction methods used commonly, such as using boiling ethanol and using perchloric acid, appear in the literature, but will not be examined here, as they focus on more difficult to extract biological systems and result in the lower recovery of metabolites [8].

All these types of extraction methods rely on large extraction volumes into which a relatively small amount of biomass (either quenched live cells or cell free extracts (CFEs)) is added, then concentrated down either via lyophilization or vacuum concentration, and the resultant material reconstituted for liquid chromatography—mass spectrometry (LC-MS) analysis. However, this additional drying-down step removes a large number of metabolites, as many do not resuspend and volatile compounds evaporate off, so important elements of the metabolome are lost [9]. There is therefore an increasing trend to use extraction methods that do not involve a drying down step, such as the method that combines methanol, water and chloroform in a 3:1:1 ratio in smaller volumes [10], so direct LC-MS analysis, without drying down, can be performed.

Each of these methods has a unique interaction with the components of the metabolome of *E. coli*, and each resulting profile will therefore be different. When using an untargeted approach to characterize whole metabolomes, it becomes necessary to maximize the number of metabolites the system can determine. While using a combination of methods is the obvious solution to this, it is more efficient to choose as few complementary methods as possible, with regard to the respective metabolites extracted, so that redundancies are removed and machine time to analyze the mass spectra of each sample is minimized.

In this paper, we therefore aim to show that, despite the additional resource cost, to obtain a high recovery and coverage of metabolites, multiple extraction methods are required, and we identify two such extraction methods that have high complementarity with respect to their profile of extracted metabolites. 

## 2. Materials and Methods

### 2.1. Generation of Cell Free Extracts

Recombinant *E. coli* BL21(DE3) clones were inoculated into 800 mL of Terrific Broth microbiological culture media in 2 L conical flasks. To generate CFEs, the cell cultures were transferred to 500 mL Nalgene centrifuge pots and spun at 6000× *g* for 10 min. This process was repeated until all cultures were reduced down to cell pellets. The pellets were weighed, and then 50 mM sodium phosphate buffer (pH 7.5) was added at 5:1 (*v*:*w*), and the cell pellets were resuspended by gentle stirring on ice. The cell suspensions were then sonicated using an MSE Soniprep 150 on 16 µm amplitude for 2 min × 4. The sonicated cell suspensions were transferred back to 500 mL Nalgene centrifuge pots and centrifuged at 13,000× *g* for 45 min and the supernatant transferred to lyophilization trays, and frozen at −24 °C. The frozen trays containing cell-free extracts were then lyophilized using Edwards Supermodulyo freeze-dryers; once complete, the powders were stored at −20 °C for analysis.

### 2.2. Extraction Methods

Sonicating was done using a Grant ultrasonic bath XUBA1 sonicator (44 kHz); vacuum concentration was performed using a Thermo Scientific (Waltham, MA, USA) Speed-Vacuum DNA130 vacuum concentrator (vacuum pressure < 13 mbar); lyophilization was performed using an Edwards EF4 freeze-dryer. 

The extraction methods detailed below used similar techniques with varying solvent compositions (Table 1).

Methods A, B and D: In total, 1 mL of extraction solvent was added to 10 mg of lyophilized CFE of each recombinant *E. coli* BL21 (DE3) clone in a low-protein-binding microcentrifuge tube (Low Protein Binding 1.5 mL microcentrifuge tube, Thermo Scientific, Waltham, MA, USA). Then, each microcentrifuge tube was vortexed vigorously for 30 s and sonicated in an ice bath for 5 min. The tubes were then further incubated on ice for 30 min before being centrifuged at 21,380 g for 10 min. The resulting supernatant was transferred into a low-protein-binding microcentrifuge tube and either (i) placed in the vacuum concentrator for 2 h at 35 °C (Code S, Table 2) or (ii) 1 mL of HPLC grade water was added, the sample was left at −80 °C until frozen and was then lyophilized overnight (Code L, Table 2). The dried samples were stored at −80 °C. Method C: In total, 1 mL of extraction solvent was added to 10 mg of lyophilized CFE of each recombinant *E. coli* BL21(DE3) clone in a low-protein-binding microcentrifuge tube. Then, each microcentrifuge tube was vortexed vigorously for 30 s and sonicated in an ice bath for 5 min. The tubes were then further incubated on ice for 30 min. Then, 0.4 mL of HPLC grade water was added, and the resultant biphasic system went through a second round of vortexing, sonication and incubation on ice, as before. After the next incubation period on ice, the tubes were centrifuged at 9503× *g* for 10 min. The upper aqueous layer of supernatant and the lower organic layer were separated into two separate microcentrifuge tubes and either (i) placed in the vacuum concentrator for 2 h at 35 °C (Code S, Table 2) or (ii) 1 mL of HPLC grade water was added, the sample was left at −80 °C until frozen and then lyophilized overnight (Code L, Table 2), respectively. The dried samples were stored at −80 °C.

Method E: In total, 0.2 mL of extraction solvent was added to 10 mg of lyophilized CFE of each recombinant *E. coli* BL21(DE3) clone in a low-protein-binding microcentrifuge tube. Then, each microcentrifuge tube was vortexed vigorously for 30 s and sonicated in an ice bath for 5 min. The tubes were then further incubated on ice for 30 min before being centrifuged at 21,380× *g* for 10 min, and the supernatant was transferred to a fresh low-protein-binding microcentrifuge tube. The samples were then stored at −80 °C. 

Method F: In total, 0.2 mL of extraction solvent was added to 10 mg of lyophilized CFE of each recombinant *E. coli* BL21(DE3) clone in a low-protein-binding microcentrifuge tube. Then, each microcentrifuge tube was vortexed vigorously for 30 s and sonicated in an ice bath for 5 min. The tubes were then further incubated on ice for 30 min before being centrifuged at 21,380× *g* for 10 min. The resulting supernatant was transferred into a low-protein-binding microcentrifuge tube and either (i) placed in the vacuum concentrator for 2 h at 35 °C (Code S, Table 2) or (ii) 1 mL HPLC grade water was added, and the samples were left at −80 °C until frozen and then lyophilized overnight (Code L, Table 2). The dried samples were stored at −80 °C. 

Method G: In total, 0.2 mL of extraction solvent was added to 10 mg of lyophilized CFE of each recombinant *E. coli* BL21(DE3) clone in a low-protein-binding microcentrifuge tube. Then, each microcentrifuge tube was vortexed vigorously for 30 s and sonicated in an ice bath for 5 min. The tubes were then further incubated on ice for 30 min before being centrifuged at 21,380× *g* for 10 min. The samples were then stored at −80 °C.

Filtration: One experiment involved filtering each sample before injection. In these cases, for non-dried down extractions, 100 μL of the sample was pipetted into a Corning Costar Spin-X centrifuge tube filter, with a cellulose acetate membrane, pore size 0.22 μm, placed inside a microcentrifuge tube and centrifuged at 2370× *g*. In total, 50 μL of the filtrate was pipetted into vials. For dried down extractions, the samples were resuspended in 100 μL of the chromatography running buffer first, then the same process followed.

### 2.3. Chromatography

The metabolite profiling of the CFEs of each recombinant *E. coli* BL21(DE3) clone was performed on a Vanquish Liquid Chromatography chromatographic separation system connected to an Orbitrap IQ-X Tribrid Mass Spectrometer (Thermo Scientific). 

CFE samples were subjected to hydrophilic liquid interaction chromatography (HILIC). The chromatographic separation was achieved using a Waters Acquity UPLC BEH amide column (2.1 × 150 mm with particle size of 1.7 μm), part no. 186004802, operating at 45 °C with a flow rate of 200 μL/min. The gradient consisted of a binary buffer system, buffer A (95% *v*/*v* water/5% *v*/*v* acetonitrile) and Buffer B (95% *v*/*v* acetonitrile/5% *v*/*v* water); both buffers contained 10 mM ammonium formate. Independent buffer systems were used for positive and negative mode acquisition; for positive mode, the pH of buffers was adjusted to 3 using 0.1% *w*/*v* formic acid, and for negative mode, the pH of buffers was adjusted to 10 using 0.1% *w*/*v* ammonium formate solution. The gradients were the same for both polarities: 95% B for 1.5 min, then linearly decreased to 50% B after a further 10.5 min, then held for 4.5 min. Then, the system was returned to the starting conditions and held for a further 4.5 min (column equilibration). The voltages applied for positive mode and negative mode were 3.5 kV and 2.5 kV, respectively. 

Injection volume used: positive mode 3 μL and negative mode 6 μL (for the experiment involving 10 mg vs. 20 mg analysis, 10 μL was used for 10 mg samples and 5 μL was used for 20 mg samples).

### 2.4. Mass Spectrometry Acquisition

The MS data were acquired using the AcquireX acquisition workflow (data dependent analysis). The MS operating parameters were as follows: MS1 mass resolution 60 K for MS2 30 K. Stepped energy (HCD) of 20, 25 and 50 was applied with a scan range of 100–1000 *m*/*z*, RF lens (%) 35, and an AGC gain intensity threshold of 2e4 25%, in the custom injection mode with an injection time of 54 ms. An extraction blank was used to create a background exclusion list and a pooled QC was used to create the inclusion list.

The HESI conditions for each 200 μL of eluent per minute were as follows: sheath gas—35, aux gas—7, sweep gas—0, ion transfer tube temperature—300 °C and vaporizer temperature—275 °C. 

### 2.5. Data Analysis

Post data processing. The HILIC positive and negative data sets were processed via Compound Discoverer 3.2 (Thermo Fisher Scientific, Hemel Hempstead, UK) according to the following settings of an Untargeted Metabolomic workflow: mass tolerance 10 ppm, maximum shift 0.3 min, alignment model adaptive curve, minimum intensity 1e6, S/N threshold 3, compound consolidation, retention time (RT) tolerance 0.3 min. Database matching was performed at the MS2 level using the mzCloud database (Thermo Scientific) with a similar index of 70% or better. Only metabolites identified via MS2 were retained. 

Quality control. Corresponding HILIC pooled QC samples were used to assess for instrumental drifts. Only metabolite features with a relative standard deviation (RSD) of 25% or less within the QC samples were retained, and this was extended to the rest of the dataset.

The total metabolites of detected data sets were combined for multivariate data analysis and trends assessments. Heat maps, principal component analysis (PCA) plots and partial least squares (PLS) data were analyzed using Metaboanalyst.

### 2.6. Experimental Steps Undertaken 

**Figure 1:**
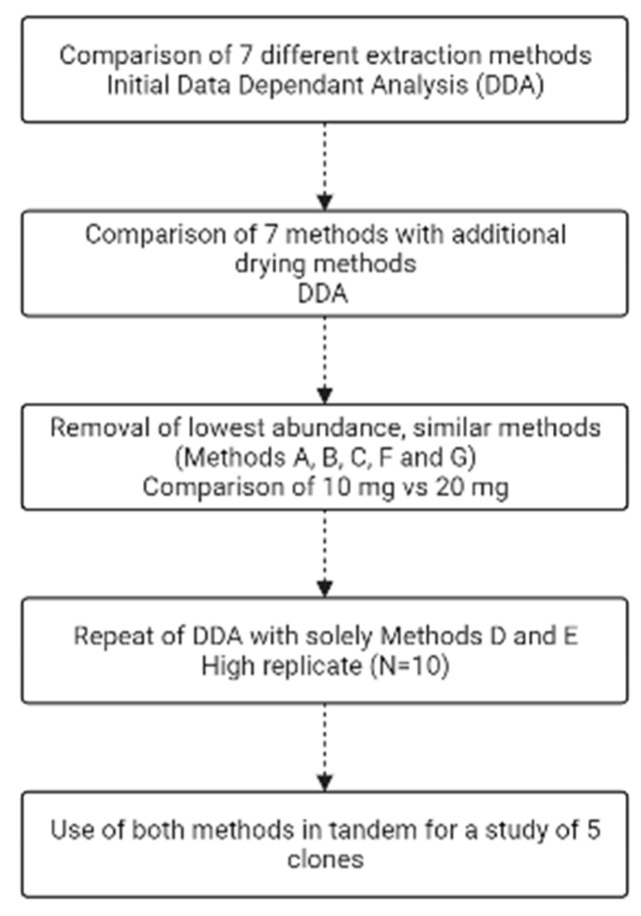
Flow chart of the experimental steps undertaken. Made using Biorender.

To asses each extraction method in order to find the optimal combination to maximize metabolite identification, a series of experiments was performed sequentially to narrow down not only (i) the best extraction methods yielding the highest number of metabolites, but also (ii) the best amount of starting biomass for each recombinant *E. coli* BL21 (DE3) clone, (iii) the method of drying down before reconstitution and (iv) whether filtration of the sample removes any metabolites (Figure 1).

## 3. Results

### 3.1. Creating the Analytical Landscape with a Range of Methods

**Figure 2:**
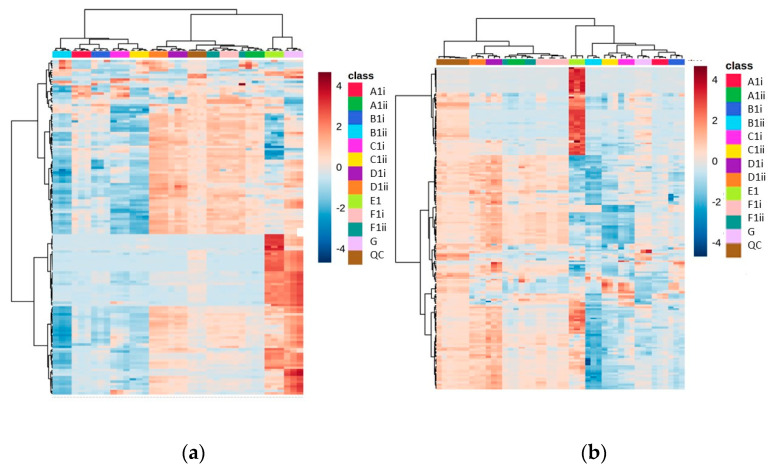
Heat maps of identified metabolite abundance from the CFE extract of a recombinant *E. coli* BL21 (DE3) clone expressing a Galactose Oxidase (Prozomix code: M3-5) for (**a**) positive mode chromatography and (**b**) negative mode chromatography. Extraction methods are shown in the class key, (i) and (ii) denote vacuum concentration and lyophilization, respectively. For positive mode, 150 features were identified (with >70% match) out of 2846 MS2 features (with <25% RSD), while for negative mode 150 features were identified (with >70% match) out of 1736 MS2 features (with <25% RSD). Columns within each class of extraction method correspond to extraction replicates (n = 3), while rows correspond to identified metabolites. The graded color scale corresponds to normalized abundance. See Figure S2 for heat maps of all MS2 features, and for a full list of identified metabolites see Metabolights ref: MTBLS7326.

Using the seven different extraction methods (Methods A–G) with the CFE of a recombinant *E. coli* BL21 (DE3) clone expressing a Galactose Oxidase (Prozomix code: M3-5), after alignment of the mass spectrometry data, 7920 total MS features (with a relative standard deviation less than 25% in the QC samples) were detected in the positive mode and 4280 total MS features in the negative mode. Further to this, 150 and 149 compounds were identified with over 70% match to the mzCloud database in positive and negative modes, respectively. The spread of compounds present over the different extraction methods revealed a clear division between those methods that do not involve drying down (Methods E and G), and the other methods. The identified features included many volatiles, such as acetylated amino acids, and this supports previous observations that show that methods without a drying down step retain volatiles. In the heat map for these data, the most abundant metabolites are uniquely grouped under Method E, and are also split from the other methods across the dendrogram, suggesting a large statistical difference to the other methods (Figure 2).

Interestingly, while Methods A, B and D showed largely similar profiles to one another (both in their chromatograms and in the identified features obtained), Method E showed more features at the end of the chromatogram (Figure S1), but had lower resolution at the start of the chromatogram compared to Methods A, B, C and D. When Method E was altered to include a drying down step (Method F), the unique features present at RTs of <8 min disappeared. All methods with drying down steps are largely similar in terms of number of features, but they vary in abundance.

Additionally, methods involving lyophilization as the drying down step appeared to have lower abundances of each feature. There was also a reduction in reproducibility between lyophilized samples (the average RSD across Methods D and F was 22.65% in vacuum-concentrated vs. 27.78% in lyophilized) for positive mode (although for negative mode it was 37.65% and 36.59%, respectively). Out of all methods involving a drying down step, Method D was shown to have the highest number of reproducible features (RSD < 25% and abundance >50,000 count) when looking at the combined identified and unidentified data (>1000 features for both lyophilized and vacuum concentration data sets in the negative mode and positive mode).

### 3.2. Comparison of 10 mg vs. 20 mg Starting Biomass (Focusing on Methods D, E, F and G) Revealed No Significant Difference between the Two Starting Masses While Filtration Resulted in Poor Reproducibility

The amount of starting biomass for each extraction was also studied, i.e., whether 10 mg of CFE would produce a comparable result to 20 mg CFE (by injecting twice the volume of the 10 mg CFE extraction compared to the 20 mg CFE extraction) and so a further experiment was conducted excluding the extraction methods, which yielded the lowest abundances of metabolites (Methods A, B and C). This also served to reinforce both the effect of drying down and the complementarity between Method E and the others.

The other factor considered was the effect of filtering the samples before they were injected. Filtering is often used to remove any particulates that are not obvious by eye, which can damage or block the chromatography column when performing such high-throughput work. 

**Figure 3:**
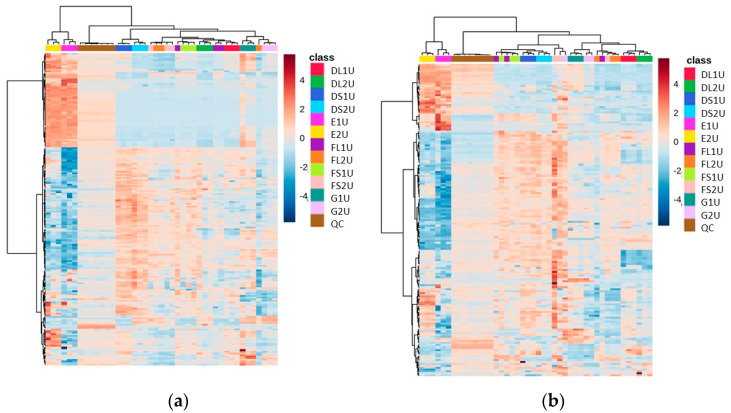
Heat maps of identified metabolite abundance from the CFE extract of a recombinant *E. coli* BL21 (DE3) clone expressing a Galactose Oxidase (Prozomix code: M3-5) for (**a**) positive mode chromatography and (**b**) negative mode chromatography. Details of starting biomass and drying down method are shown in Table 2. For positive mode, 120 features were identified (with >70% match) out of 2801 MS2 features (with <25% RSD), while for negative mode 84 features were identified (with >70% match) out of 2171 MS2 features (with <25% RSD). Columns within each class of extraction method correspond to extraction replicates (n = 3), rows correspond to identified metabolites. Graded color scale corresponds to normalized abundance. See Figure S3 for heat maps of all MS2 features, and for a full list of identified metabolites see Metabolights ref: MTBLS7326.

The heat map of metabolite abundances for Methods D and E, with vacuum concentration as the drying down method in Method D, revealed again that two distinct regions appear in the analytical space (where we see one section of metabolites that have uniquely high abundance in Method E and another section that corresponds to methods with drying steps), revealing the complementarity between these methods (Figure 3). 

In this experiment, of the dried down methods, Method D had a higher number of features present (>1500 features in the positive mode and >1100 in the negative mode) at an abundance over 50,000, and with an RSD less than 25% (Table 3). The lyophilized replicates of Method D produced fewer reproducible features, and so it was decided that vacuum concentration would be used as the preferred drying down method. Method G, while showing a high number of hits in the negative mode, had a very high RSD (>35% in most variations, Table S1), and even though it was more reproducible in the unfiltered negative mode than Method E, there was much less coverage of the metabolites shown in Method E that were complementary to Method D (Figure 3).

When comparing 10 mg and 20 mg starting biomass extractions, the average RSDs for these two conditions were very similar (29.14% and 26.95%, respectively) in the positive mode. However, 10 mg gave less variability than 20 mg (RSDs of 38.52% and 49.38% respectively) when in the negative mode. This, combined with the fact that more features are identified above an abundance of 50,000 counts in the majority of extraction methods using 10 mg (Table 3), suggests that using 10 mg will not only be a more useful amount in terms of minimizing the wastage of precious CFE, but also provides more reproducible results with better extraction. 

The other feature of this set of experiments was the comparison between using filtered and unfiltered material post-reconstitution in the chromatography running buffer. It was concluded that the filtering process introduced an increase in variability (Table 3), particularly in the negative mode analysis. In addition, there was also a reduction in the abundance of features present.

### 3.3. High Replicate Experiment Focusing on Methods D and E Demonstrated Stark Complementarity

**Figure 4:**
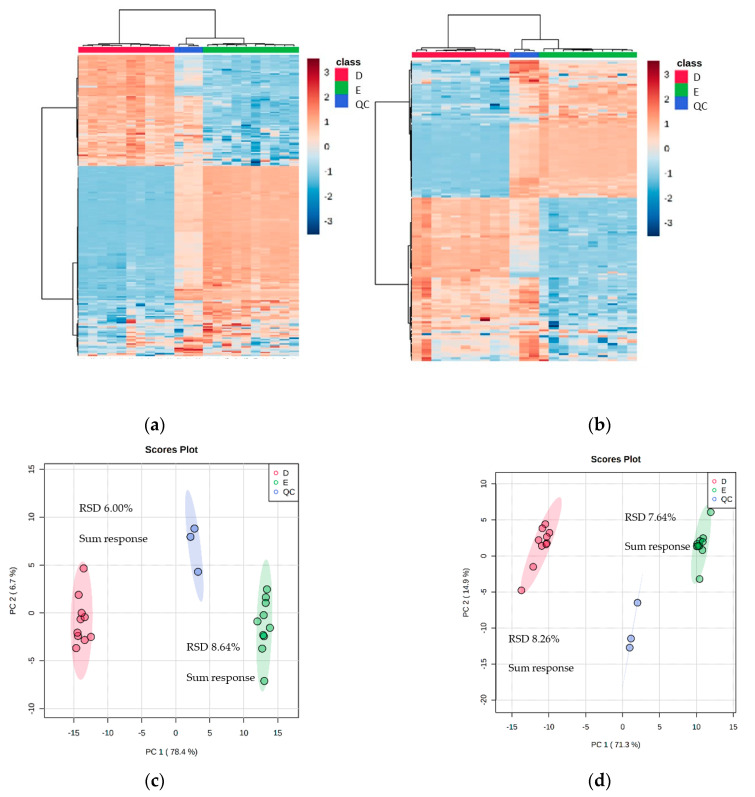
Heat maps and PCA plots of identified metabolite abundance from the CFE extract of a recombinant *E. coli* BL21 (DE3) clone expressing a Galactose Oxidase (Prozomix code: M3-5) for (**a**,**c**) positive mode and (**b**,**d**) negative mode chromatography, respectively. For positive mode, 211 features were identified (with >70% match) out of 2580 MS2 features (with <25% RSD), while for negative mode 151 features were identified (with >70% match) out of 1864 MS2 features (with <25% RSD). Columns within each class of extraction method correspond to extraction replicates (n = 10), rows correspond to identified metabolites. The graded color scale corresponds to normalized abundance. The RSD values of the sum responses are shown for PCA plots. See Figure S4 for heat maps of all MS2 features, and for a full list of identified metabolites see Metabolights ref: MTBLS7326.

To ascertain the effectiveness and robustness of a dual extraction methodology, an n = 10 experiment was done using Method D (with vacuum concentrating) and Method E for the recombinant *E. coli* BL21 (DE3) clone expressing a Galactose Oxidase (M3-5). The mass spectrometry data revealed that there were 2580 features in the positive mode and 1864 in the negative mode (including 211 and 151 features with over 70% match to the mzCloud database). The heat maps for the two selected extraction methods showed distinct quadrants of metabolite abundance, highlighting the complementarity between the extraction methods (Figure 4). 

### 3.4. Utilizing a Dual Extraction Methodology Revealed Significant Differences between Recombinant E. coli BL21(DE3) Clones

Four recombinant *E. coli* BL21 (DE3) clones were chosen that expressed enzymes of different functionality (a Galactose Oxidase (M3-5), two cytochrome P450s from different classes (Prozomix codes: 7-8 and HA1) and a ketoreductase (Prozomix code: KR271), plus a control with an empty pET-28a plasmid vector (code: C)). It was hypothesized that these four recombinant *E. coli* BL21 (DE3) clones would exhibit large differences in their metabolome, and thus be spread across the analytical space when the metabolites present were analyzed using PCA. Using these four different recombinant *E. coli* BL21 (DE3) clones, it was shown that significant differences could be shown between each clone, in terms of which metabolites are present at high abundances. Using a higher number of replicates also reduced the global RSD across each recombinant *E. coli* BL21 (DE3) clone (Table 4).

**Figure 5:**
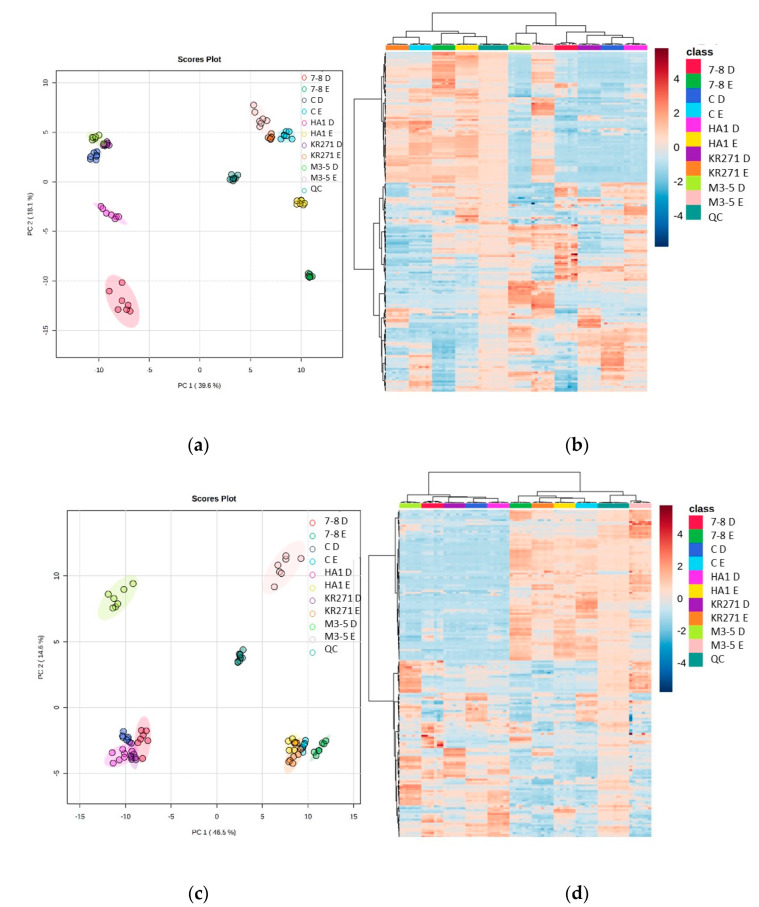
Heat maps and PCA plots of identified metabolite abundance from the CFE extracts of all four recombinant *E. coli* BL21 (DE3) clones (a Galactose Oxidase (Prozomix code: M3-5), two cytochrome P450s from different classes (Prozomix codes: 7-8 and HA1), a ketoreductase (Prozomix code: KR271) and a control with an empty pET-28a plasmid vector (code: C)) for (**a**,**c**) positive mode and (**b**,**d**) negative mode chromatography, respectively. In positive mode, 187 compounds were identified (with >70% match) out of 3041 MS2 features (with <25% RSD), and in negative mode 172 compounds were identified (with >70% match) out of 3200 MS2 features (with <25% RSD). Columns within each class of extraction method correspond to extraction replicates (n = 3), rows correspond to identified metabolites. Graded color scale corresponds to normalized abundance. The RSD values of the sum responses are shown in the PCA plots. See Figure S5 for heat maps of all MS2 features, and for a full list of identified metabolites see Metabolights ref: MTBLS7326.

The heat maps show further evidence that there is a large degree of complementarity between each extraction method. Furthermore, they reveal that each recombinant *E. coli* BL21 (DE3) clone has clusters of metabolites that feature uniquely prominently in that clone. This provides good evidence that the recombinant enzymes in each clone have a unique effect on the metabolome of *E. coli*, and therefore this combination of extraction methods will be able to identify novel metabolites that appear when undertaking a high-throughput analysis of different clones. Two recombinant *E. coli* BL21 (DE3) clones, in particular (M3-5 and 7-8), appear to exhibit more significant differences to the other clones (they are split from the others across PC2 (PC1 splitting is mainly a feature of the difference between extraction methods (Figure 5)).

The data not only reveal the differences between these recombinant *E. coli* BL21(DE3) clones, but the PLS analysis allows us to see which individual features in the MS explained more of the variance in the data. This analysis was performed separately for each extraction method across all four recombinant *E. coli* BL21 (DE3) clones. When looking at the features with a VIP score of more than 1.2, for the unassigned features, there are more than 1100 features in the positive mode for each method and 1400 in the negative mode. The overlap of these features also further shows how complementary the methods are, with 50% of the features that account for most variance appearing uniquely in the separate extraction methods (Table 5).

**Figure 6:**
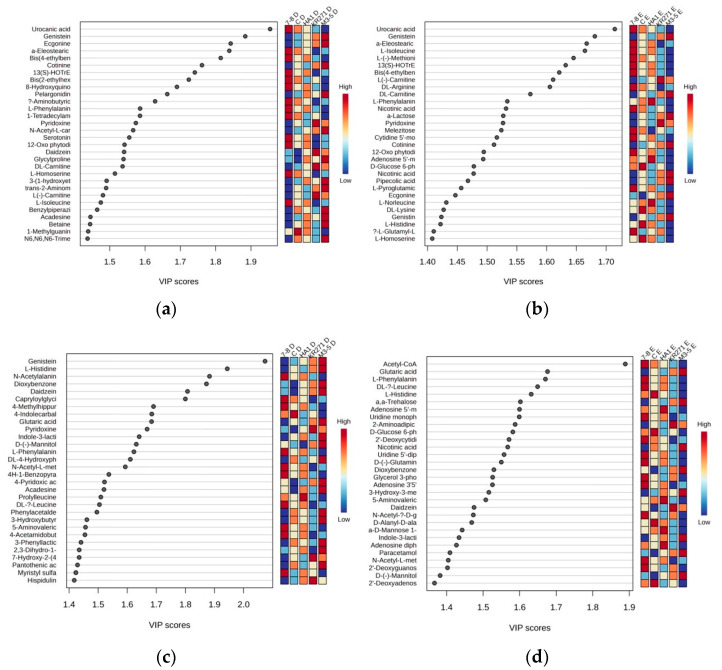
Partial least square data showing important features within each extraction for the top 30 metabolites. (**a**) Positive mode using Method D, (**b**) positive mode using Method E, (**c**) negative mode using Method D and (**d**) negative mode using Method E. For a full list of metabolites with a VIP score above 1.2, see supplementary information (Table S2).

For the identified metabolites, there were a total of 77 features across both extractions in the positive mode and 76 features from the negative mode. Many of these features were primary metabolites, such as acetyl CoA (which had a very high score of nearly 1.9 relative to the next highest, genistein, which was just under 1.7 in the negative mode for Method E) and numerous primary and acetylated amino acids, suggesting that the recombinant enzyme induces stress in *E. coli* [11], which is common in bacteria used in protein expression systems [12]. Interestingly, in the positive mode for both extractions, uraconic acid, an intermediate in the catabolism of amino acid L-histidine, appeared to be the number one factor accounting for the variance in the data (Figure 6).

## 4. Discussion

When comparing Methods D and E to one another, one of the major differences is within the chromatography. Method E showed much greater density of metabolites at a RT later than 8 min, with lower resolution of peaks in the earlier half, whereas Method D had much better resolution of chromatography before 8 min. One of the most likely reasons for the distortion of the chromatography in Method E is that the extraction solvent is out of phase with the running buffer, as there is no drying down and reconstitution in the running buffer with this extraction method. The methanol and chloroform contents of Method E samples therefore interfere with the HILIC chromatography, which probably accounts for the broadening of the peaks due to the phase incompatibility of the extraction solvent and the running buffer [9]. This slight interference, however, is greatly outweighed by the extra information that Method E provides about the metabolome of the recombinant *E. coli* clones. The column and ionization source have not shown any damage caused by the solvent loads, as the percentage of chloroform is not high enough to cause damage (i.e., 0.8 µL diluted in a flow rate of 200 µL/min). To date, this method has been run over a 6-month period, and there has been no permanent distortion of chromatography or degradation of performance. 

One of the many advantages of this system is that there is no need for any extra normalization of each sample, as the normalization is built into the set up when weighing out the CFE, as opposed to the post-acquisition normalization methods often used in quantitative metabolomics [13]. 

The majority of metabolites revealed to be significantly different across the four recombinant *E. coli* clones were metabolites heavily involved in primary metabolism. While it was hypothesized that these differences were due solely to the recombinant enzymes added, it is unwise to make inferences about the exact causes of the differences in the levels of each metabolite in this study of four recombinant *E. coli* clones, as each clone was grown in different batches of medium and so will likely have had different compositions of starting nutrients [14]. It is enough for the purpose of this study to show that a large number of metabolites can be detected in either of the methods chosen, and that the methods show complementarity. 

The nature of the metabolites identified in Method E across the recombinant *E. coli* clones (i.e., largely common primary metabolites that are vital for survival) poses an interesting problem. While useful when looking at the gross differences between recombinant *E. coli* clones, it is less useful for identifying individual novel metabolites due to unique recombinant enzymes, as the primary metabolites are much more likely to be common across different clones. When using PCA to analyze the data, the risk is that potential individual metabolites present at a high level (indicative of a novel metabolic pathway) will be lost in the data decomposition [15]. This can potentially be mitigated by looking at unique high-abundance features between recombinant *E. coli* clones on the raw data level in conjunction with carrying out multivariant analysis methods. Further studies will be required, however, as it is clear from the PLS data that some metabolites that are a large source of variance between recombinant *E. coli* clones are primary metabolites—for example, acetyl CoA and assorted amino acids (Figure 6).

## 5. Conclusions

Methods D and E have been shown to be complementary to each other, and have also been shown to cover a large range of metabolites and MS2 features. The further optimization of these methods has shown that it is possible to produce very similar results with high reproducibility using 10 mg of CFE. Vacuum-concentrating the extracted solution for Method D also reveals better reproducibility and a greater number of features. The combination of these methods was also very effective at showing differences between recombinant *E. coli* clones, thus proving its use as a tool for observing the changing metabolome of *E. coli* in response to the recombinant enzymes they are expressing. Further research needs to be done into the use of programs such as Compound Discoverer to elucidate the structures of some of the unknown features that are appearing, and so increase the proportion of compounds that we can identify. This will also help us to establish the exact synthetic pathways involved between a variety of different recombinant *E. coli* clones, and will be applicable to other bacterial systems. 

## Figures and Tables

**Table 1:** Extraction solvent composition. Method C is a two-stage process, as indicated by the use of parentheses.

Method	Solvents	Ratio
A	Methanol:Water	4:1
B	Methanol:Chloroform	3:1
C	Chloroform:Methanol (:Water)	2:1 (:1.2)
D/G	Acetonitrile:Water	4:1
E/F	Methanol:Water:Chloroform	3:1:1

**Table 2:** Nomenclature for comparison of starting biomass and filtration pre-injection vs. no filtration for extraction methods. (**a**) Drying down methods and (**b**) non-drying down methods.

		Filtered Before Injection		Unfiltered Before Injection	
Extraction method	Starting Biomass (mg)	Vacuum Concentration	Lyophilization	Vacuum Concentration	Lyophilization
D	10	DS1F	DL1F	DS1U	DL1U
	20	DS2F	DL2F	DS2U	DL2U
F	10	FS1F	FL1F	FS1U	FL1U
	20	FS2F	FL2F	FS2U	FL2U
(**a**)					
Extraction Method	Starting Biomass (mg)	Filtered Before Injection		Unfiltered Before Injection	
E	10	E1F		E1U	
	20	E2F		E2U	
G	10	G1F		G1U	
	20	G2F		G2U	
(**b**)					

**Table 3:** Unique features present above a count of 50,000 that had an RSD < 25% across each replicate for positive mode and negative mode.

Positive Mode		Negative Mode	
Extraction	Features	Extraction	Features
DL1F	840	DL1F	1300
DL1U	787	DL1U	1201
DL2F	73	DL2F	1134
DL2U	742	DL2U	1106
DS1F	1131	DS1F	1669
DS1U	1206	DS1U	1703
DS2F	133	DS2F	1577
DS2U	955	DS2U	1515
E1F	68	E1F	856
E1U	140	E1U	658
E2F	573	E2F	1418
E2U	675	E2U	1009
FL1F	506	FL1F	1327
FL1U	355	FL1U	929
FL2F	710	FL2F	571
FL2U	536	FL2U	532
FS1F	900	FS1F	1386
FS1U	843	FS1U	1195
FS2F	947	FS2F	956
FS2U	81	FS2U	1200
G1F	272	G1F	558
G1U	886	G1U	1326
G2F	214	G2F	1170
G2U	165	G2U	1461
QC	1325	QC	2115

**Table 4:** Average RSD for each extraction method used for each recombinant *E. coli* BL21 (DE3) clone under positive and negative modes.

Clone/Extraction Method	Pos Average RSD	Neg Average RSD
7-8 D	31.01	41.29
7-8 E	16.47	32.98
C D	18.59	28.54
C E	16.24	25.67
HA1 D	28.14	33.30
HA1 E	18.09	26.72
KR271 D	19.11	25.70
KR271 E	17.62	31.32
M3-5 D	21.84	39.24
M3-5 E	27.75	45.29
Average	21.49	33.00

**Table 5:** Table showing the number of MS2 features with a VIP score >1.2 appearing uniquely in each extraction method.

Extraction Method	Positive Mode	Negative Mode
D	367	463
E	340	528

## Data Availability

The raw data presented in this study are available in the EMBL-EBIMetaboLights database at www.ebi.ac.uk/metabolights/MTBLS7326 (Data uploaded on 12 June 2023) with the reference MTBLS7326.

## Supplementary Materials

The following supporting information can be downloaded at: https://www.mdpi.com/article/10.3390/metabo13091010/s1, Figure S1: Chromatograms of metabolites extracted from the CFE of a recombinant *E. coli* BL21(DE3) clone expressing a Galactose Oxidase (Prozomix code: M3-5; Figure S2: Heat maps of metabolite abundance from the CFE extract of a recombinant *E. coli* BL21(DE3) clone expressing a Galactose Oxidase (Prozomix code: M3-5). Extraction methods shown in the key, (i) and (ii) denote vacuum concentration and lyophilization, respectively; Figure S3: Heat maps of metabolite abundance from the CFE extract of a recombinant *E. coli* BL21(DE3) clone expressing a Galactose Oxidase (Prozomix code: M3-5). Details of starting biomass and drying down method were as according to Table 2; Figure S4: Heat maps and PCA plots of metabolite abundance from the CFE extract of a recombinant *E. coli* BL21(DE3) clone expressing a Galactose Oxidase (Prozomix code: M3-5); Figure S5: Heat maps and PCA plots of metabolite abundance from the CFE extracts of all four recombinant *E. coli* BL21(DE3) clones (a Galactose Oxidase (Prozomix code: M3-5), two cytochrome P450s from different classes (Prozomix codes: 7-8 and Ha1), a ketoreductase (Prozomix code: KR271) and a control with an empty pET-28a plasmid vector (code: C); Table S1: Table showing the average RSDs for each extraction method from Section 3.2; Table S2: Table showing all features from the four recombinant *E. coli* BL21(DE3) clones which have a significant VIP score (greater than 1.2) explaining the variance in the PLS-DA.
