# Supplementary material for: In Search of Complementary Extraction Methods for Comprehensive Coverage of the Escherichia coli Metabolome
Source: Metabolites. 2023 Sep 14;13(9):1010. doi: 10.3390/metabo13091010 (PMC10535102; doi:10.3390/metabo13091010)
Supplement: Supplementary file 1 [file metabolites-13-01010-s001.zip › metabolites-2573714-supplementary.pdf]

metabolites

Supplementary Materials

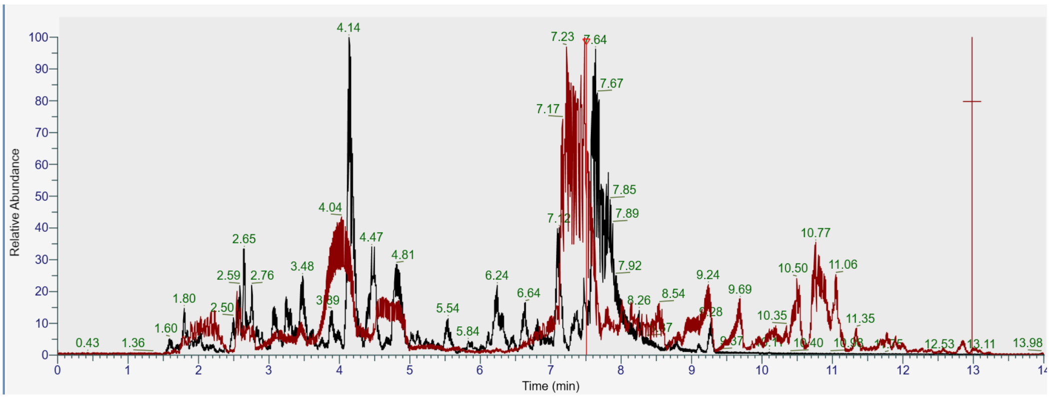

(a)

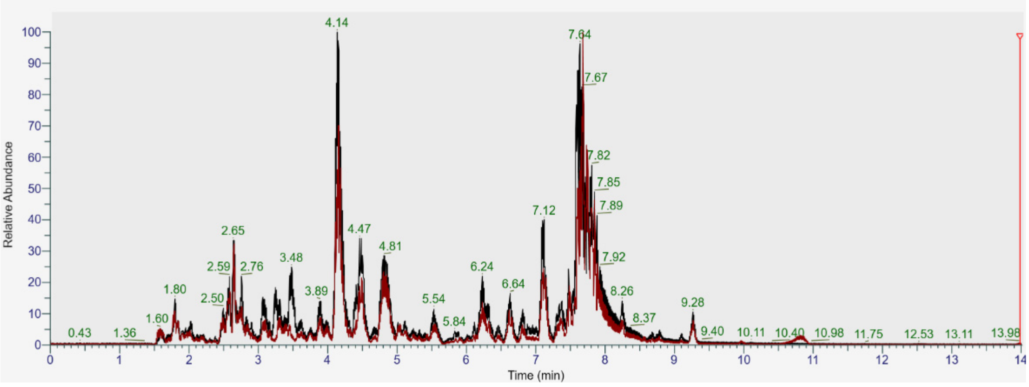

(b)

Figure S1. Chromatograms of metabolites extracted from the CFE of a recombinant *E. coli* BL21(DE3) clone expressing a Galactose Oxidase (Prozomix code: M3-5). (a) Method E (red) and Method D (black) and (b) Method F (red) and Method D (black). HILIC chromatography was used in positive mode.

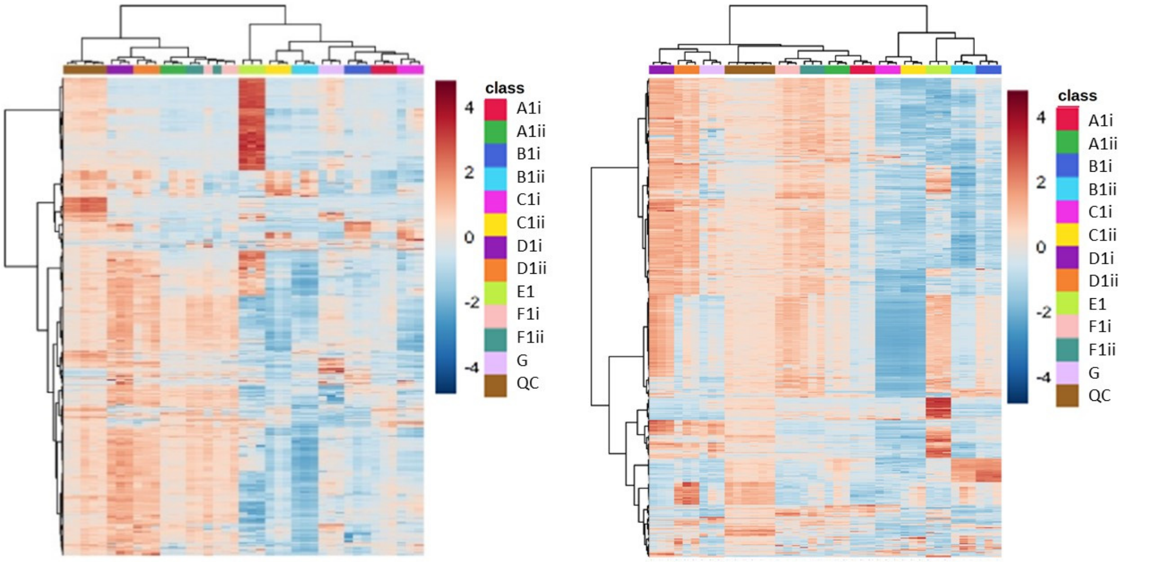

(a) **(b)**

Figure S2. Heat maps of metabolite abundance from the CFE extract of a recombinant *E. coli* BL21(DE3) clone expressing a Galactose Oxidase (Prozomix code: M3-5) for (a) positive mode chromatography and (b) negative mode chromatography for all MS2 features. Extraction methods shown in the key, i) and ii) denote vacuum concentration and lyophilization, respectively. There were 2846 MS2 features (with < 25% RSD) for positive mode and 1736 MS2 features (with < 25% RSD) for negative mode. Columns within each class of extraction method correspond to extraction replicates (n=3), rows correspond to MS2 features. The graded colour scale corresponds to normalized abundance.

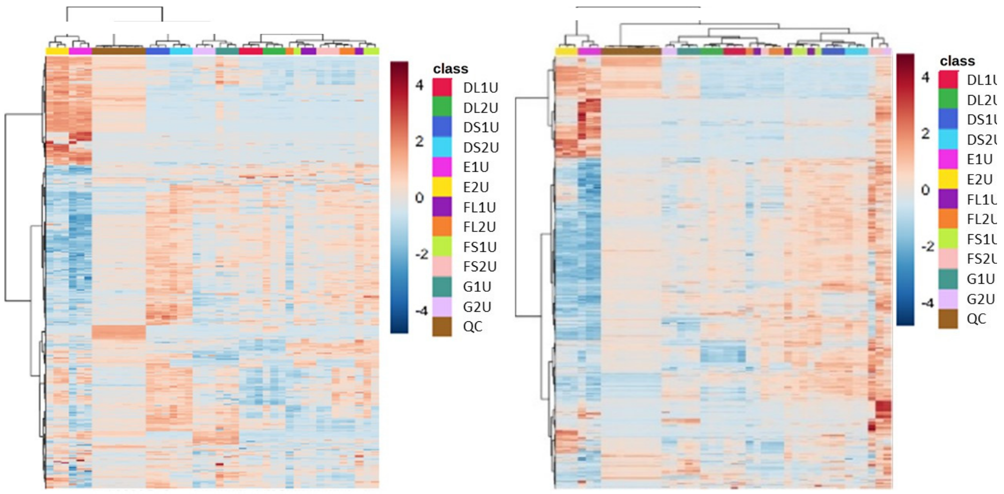

(a) **(b)**

Figure S3. Heat maps of metabolite abundance from the CFE extract of a recombinant *E. coli* BL21(DE3) clone expressing a Galactose Oxidase (Prozomix code: M3-5) for (a) positive mode chromatography and (b) negative mode chromatography for all MS2 features. Details of starting biomass and drying down method were as according to Table 2. There were 2801 MS2 features (with < 25% RSD) for positive mode and 2171 MS2 features (with < 25% RSD) for negative mode. Columns within each class of extraction method correspond to extraction replicates (n=3), rows correspond to MS2 features. The graded colour scale corresponds to normalized abundance.

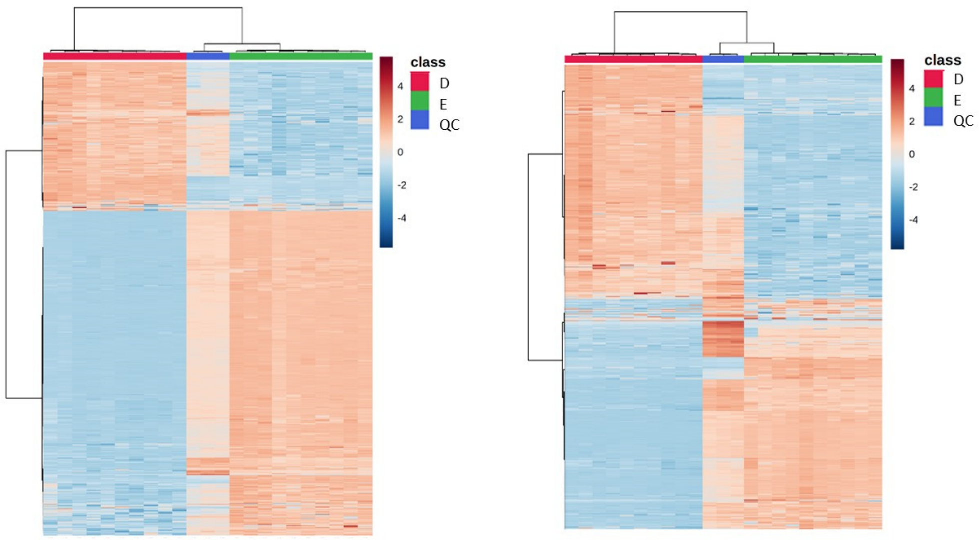

(a) **(b)**

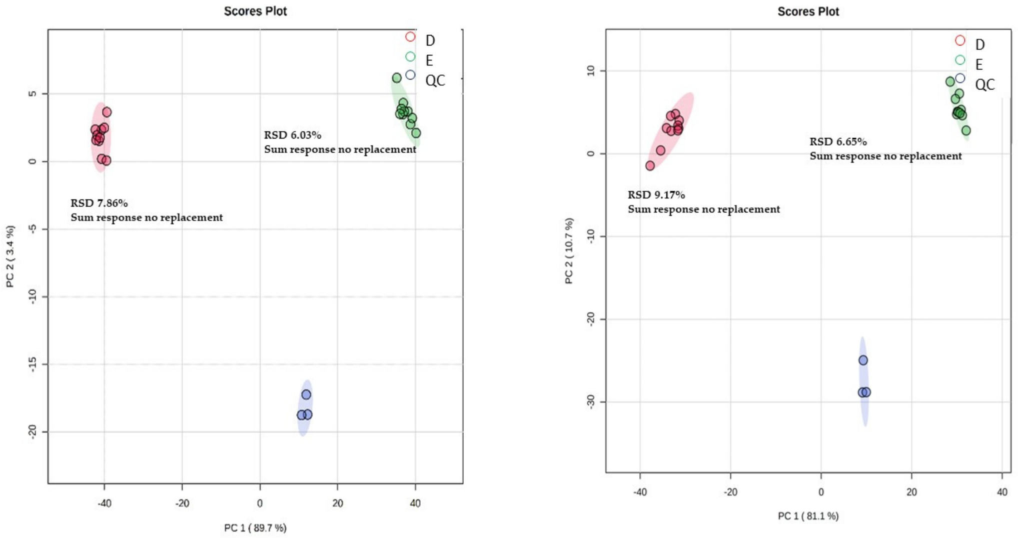

(c) **(d)**

Figure S4. Heat maps and PCA plots of metabolite abundance from the CFE extract of a recombinant *E. coli* BL21(DE3) clone expressing a Galactose Oxidase (Prozomix code: M3-5) for (a and c) positive mode and (b and d) negative mode chromatography, respectively, for all MS2 features. There were 2580 MS2 features (with < 25% RSD) for positive mode and 1864 MS2 features (with < 25% RSD) for negative mode. Columns within each class of extraction method correspond to extraction replicates (n=10), rows correspond to MS2 features. The graded colour scale corresponds to normalized abundance. The RSD values of the sum responses are shown for PCA plots.

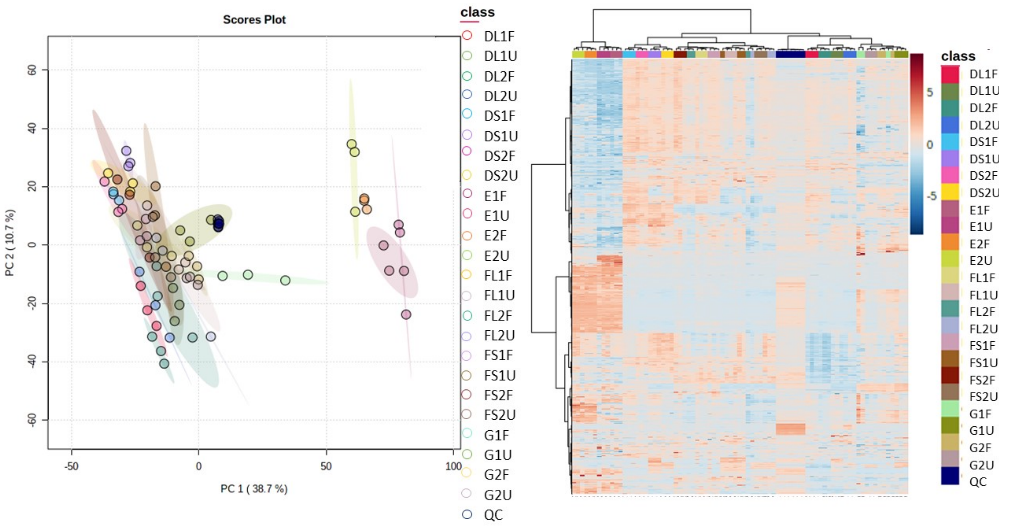

(a) **(b)**

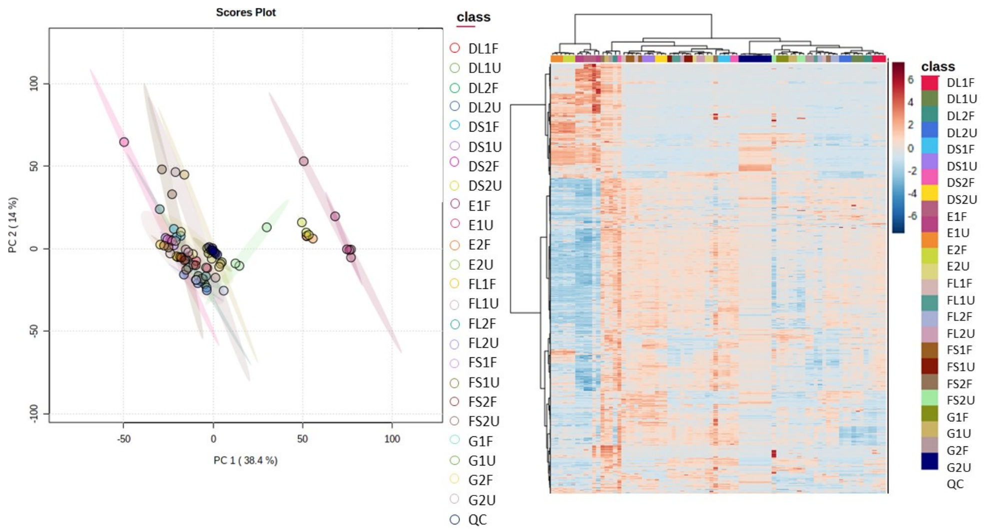

(c) **(d)**

Figure S5. Heat maps and PCA plots of metabolite abundance from the CFE extracts of all four recombinant *E. coli* BL21(DE3) clones (a Galactose Oxidase (Prozomix code: M3-5), two cytochrome P450s from different classes (Prozomix codes: 7-8 and Ha1), a ketoreductase (Prozomix code: KR271) and a control with an empty pET-28a plasmid vector (code: C) for (a and c) positive mode and (b and d) negative mode chromatography, respectively, for all MS2 features. There were 3041 MS2 features (with < 25% RSD) for positive mode and 3200 MS2 features (with < 25% RSD) for negative mode. Columns within each class of extraction method correspond to extraction replicates (n=3), rows correspond to identified metabolites. The graded colour scale corresponds to normalized abundance.

Table S1. Table showing the average RSDs for each extraction method from section 3.2 in the (a) negative mode (with filtration step) (b) negative mode (without filtration step) (c) positive mode (with filtration step) and (d) positive mode (without filtration step). RSDs were calculated from the top 50, the median 50 and the bottom 50 compounds with respect to abundance and in addition, the maximum RSD and minimum RSD for each method are displayed.

Neg Av RSD	DL1F	DL2F	EF1	EF2	DS1F	DS2F	G1F	G2F	QC
Top 50	19.88	54.35	59.29	17.7	18.05	52.11	40.90	73.96	6.83
Middle 50	27.55	51.43	86.39	40.3	18.87	48.31	58.43	75.56	10.48
Bottom 50	33.12	115.94	136.88	20.47	20.01	149.04	42.79	89.13	8.68
Max RSD	172.53	172.99	170.94	172.01	170.3	169.25	172.77	170.97	18.99
Min RSD	0.95	0.98	3.43	0.38	0.51	2.95	0.61	1.78	1.71

(a)

Neg Av. RSD	DL1U	DL2U	E1U	E2U	DS1U	DS2U	G1U	G2U	QC
Top 50	20.06	28.07	51.65	15.05	13.14	18.61	16.39	54.94	6.83
Middle 50	25.88	29.08	56.61	31.27	17.42	21.61	22.52	53.11	10.48
Bottom 50	13.43	15.11	83.16	22.6	17.08	22.3	19.23	152.06	8.68
Max. RSD	170.7	154.23	172.26	166.4	169.75	171.57	167.37	172.26	18.99
Min. RSD	0.23	0.38	1	0.25	0.45	0.47	0.28	0.78	1.71

(b)

Pos-Av RSD	DL1F	DL2F	E1F	E2F	DS1F	DS2F	G1F	G2F	QC
Top 50	8.59	12.63	38.6	12.24	5.65	10.24	36.43	27.37	6.75

Middle 50	22.58	36.12	38.56	21.65	17.66	18.96	49.16	20.40	8.56
Bottom 50	15.19	6.34	26.92	17.48	9.04	6.93	20.93	32.90	6.72
Max. RSD	164.6	160.3	171.7	169.3	170.2	168.5	170.94	171.31	24.93
Min. RSD	0.13	0.57	0.37	0.32	0.15	0.27	1.11	0.81	0.9

(c)

Pos Av. RSD	DL1U	DL2U	E1U	E2U	DS1U	DS2U	G1U	G2U	QC
Top 50	8.79	13.75	35.76	19.86	5.23	10.59	16.14	11.49	6.75
Middle 50	24.98	27.55	39.19	32.54	9.87	14.19	31.72	23.30	8.56
Bottom 50	6.32	16.09	34.16	15.56	6.4	6.62	19.95	6.69	6.72
Max. RSD	169.6	163.1	172.2	172	156.9	163.5	169.30	155.94	24.93
Min. RSD	0.29	0.22	0.33	0.22	0.16	0.27	0.47	0.26	0.9

(d)

Table S2. Table showing all features from the four recombinant *E. coli* BL21(DE3) clones which have a significant VIP score (greater than 1.2) explaining the variance in the PLS-DA. In the positive mode there are 58 metabolites shown for extraction Method D and 63 for extraction Method E; in the negative mode there are 48 for extraction Method D and 58 for extraction Method E.

**Positive Mode Extraction D**		**Positive Mode Extraction E**		**Negative Mode Extraction D**		**Negative Mode Extraction E**	
**Metabolite**	**VIP score**	**Metabolite**	**VIP score**	**Metabolite**	**VIP score**	**Metabolite**	**VIP score**
Urocanic acid	1.95	Urocanic acid	1.71	Genistein	2.07	Acetyl-CoA	1.89
Genistein	1.88	Genistein	1.68	L-Histidine	1.94	Glutaric acid	1.68
Ecgonine	1.84	a-Eleostearic acid	1.67	N-Acetylalanine	1.88	L-Phenylalanine	1.67
a-Eleostearic acid	1.84	L-Isoleucine	1.66	Dioxybenzone	1.87	DL-Leucine	1.65
Bis(4-ethylbenzylidene)sorbitol	1.81	L-(-)-Methionine	1.65	Daidzein	1.81	L-Histidine	1.63
Cotinine	1.76	13(S)-HOTrE	1.63	Capryloylglycine	1.80	a,a-Trehalose	1.60
13(S)-HOTrE	1.74	Bis(4-ethylbenzylidene)sorbitol	1.62	4-Methylhippuric acid	1.69	Adenosine 5'-monophosphate	1.60
Bis(2-ethylhexyl) amine	1.72	L(-)-Carnitine	1.61	4-Indolecarbaldehyde	1.68	Uridine monophosphate (UMP)	1.60
8-Hydroxyquinoline	1.69	DL-Arginine	1.61	Glutaric acid	1.68	2-Aminoadipic acid	1.59
Pelargonidin	1.66	DL-Carnitine	1.57	Pyridoxine	1.67	D-Glucose 6-phosphate	1.58
Aminobutyric acid (GABA)	1.63	L-Phenylalanine	1.53	Indole-3-lactic acid	1.64	2'-Deoxycytidine 5'-monophosphate (dCMP)	1.57
L-Phenylalanine	1.59	Nicotinic acid adenine dinucleotide	1.53	D-(-)-Mannitol	1.63	Nicotinic acid	1.57
1-Tetradecylamine	1.59	a-Lactose	1.53	L-Phenylalanine	1.62	Uridine 5'-diphosphate (UDP)	1.56
Pyridoxine	1.57	Pyridoxine	1.53	DL-4-Hydroxyphenyllactic acid	1.61	D-(-)-Glutamine	1.55
N-Acetyl-L-carnosine	1.57	Melezitose	1.52	N-Acetyl-L-methionine	1.59	Dioxybenzone	1.53
Serotonin	1.56	Cytidine 5'-monophosphate (hydrate)	1.52	4H-1-Benzopyran-4-one, 6-D-glucopyranosyl-2,3-dihydro-5,7-dihydroxy-2-(4-hydroxyphenyl)-, (S)-	1.54	Glycerol 3-phosphate	1.53

12-Oxo phytodienoic acid	1.54	Cotinine	1.51	4-Pyridoxic acid	1.52	Adenosine 3'5'-cyclic monophosphate	1.53
Daidzein	1.54	12-Oxo phytodienoic acid	1.49	Acadesine	1.52	3-Hydroxy-3-methylglutaric acid	1.52
Glycylproline	1.54	Adenosine 5'-monophosphate	1.49	Prolylleucine	1.51	5-Aminovaleric acid	1.51
DL-Carnitine	1.54	D-Glucose 6-phosphate	1.48	DL-Leucine	1.50	Daidzein	1.48
L-Homoserine	1.51	Nicotinic acid	1.48	Phenylacetaldehyde	1.50	N-Acetyl-D-glucosamine 1-phosphate	1.47
3-(1-hydroxyethyl)-2,3,6,7,8,8a-hexahydro-pyrrolo[1,2-a]pyrazine-1,4-dione	1.49	Pipecolic acid	1.47	3-Hydroxybutyric acid	1.46	D-Alanyl-D-alanine	1.47
trans-2-Aminomethyl-1-cyclohexanol	1.49	L-Pyroglutamic acid	1.46	5-Aminovaleric acid	1.46	a-D-Mannose 1-phosphate	1.44
L(-)-Carnitine	1.48	Ecgonine	1.45	4-Acetamidobutanoic acid	1.45	Indole-3-lactic acid	1.43
L-Isoleucine	1.47	L-Norleucine	1.43	3-Phenyllactic acid	1.44	Adenosine diphosphate ribose	1.43
Benzylpiperazine	1.46	DL-Lysine	1.43	2,3-Dihydro-1-benzofuran-2-carboxylic acid	1.43	Paracetamol	1.41
Acadesine	1.45	Genistin	1.42	7-Hydroxy-2-(4-hydroxyphenyl)-4-oxo-3,4-dihydro-2H-chromen-5-yl-D-glucopyranoside	1.43	N-Acetyl-L-methionine	1.40
Betaine	1.44	L-Histidine	1.42	Pantothenic acid	1.43	2'-Deoxyguanosine 5'-monophosphate (dGMP)	1.40
1-Methylguanine	1.44	L-Glutamyl-L-glutamic acid	1.41	Myristyl sulfate	1.42	D-(-)-Mannitol	1.38
N6,N6,N6-Trimethyl-L-lysine	1.44	L-Homoserine	1.41	Hispidulin	1.42	2'-Deoxyadenosine 5'-monophosphate (dAMP)	1.37
Pyridoxal	1.44	Glycylproline	1.40	Adenine	1.40	S-Lactoylglutathione	1.36
6-Methylquinoline	1.43	Stearamide	1.39	D-(-)-Glutamine	1.40	Uridine 5'-diphosphoglucuronic acid	1.35
[3-({3-[(Cyclopropylmethyl)amino]-3-	1.41	Daidzin	1.39	2-Anisic acid	1.39	Acadesine	1.34

oxetanyl}methyl)-1,2-oxazol-5-yl]methanol							
Anabasine	1.41	L-2-Aminoadipic acid	1.38	4-Methylbenzotriazole	1.37	Phenylacetaldehyde	1.33
Daidzin	1.40	2-Amino-4-cresol	1.38	2-(Acetylamino)hexanoic acid	1.36	Myristyl sulfate	1.33
Bethanechol	1.40	Guvacoline	1.38	Paracetamol	1.36	3-Phenyllactic acid	1.33
DL-Stachydrine	1.38	Pyridoxal	1.36	6-Hydroxycaproic acid	1.33	2-(Acetylamino)hexanoic acid	1.32
Genistin	1.38	D-(+)-Pipecolinic acid	1.34	Picolinic acid	1.31	Genistein	1.32
Pipecolic acid	1.37	N3,N4-Dimethyl-L-arginine	1.33	2'-Deoxyadenosine 5'-monophosphate (dAMP)	1.30	Riboflavin	1.31
Prolylleucine	1.36	N-Acetyl-L-carnosine	1.32	Guanine	1.30	4-Methylhippuric acid	1.31
L-Pyroglutamic acid	1.36	Anabasine	1.31	cis-Aconitic acid	1.30	2-Hydroxycinnamic acid	1.30
Tyramine	1.35	Daidzein	1.31	Glycitein	1.30	O-Acetylserine	1.29
Norharman	1.35	Didecyldimethylammonium	1.30	Mesalamine	1.30	Phosphoenolpyruvic acid	1.28
Acetophenone	1.35	Caprolactam	1.30	2-Hydroxyvaleric acid	1.27	Dodecyl sulfate	1.28
4-Hydroxybenzaldehyde	1.35	Betaine	1.29	Nicotinic acid	1.25	D-Hydroxyglutaric acid	1.27
Levetiracetam	1.34	[3-({3-[(Cyclopropylmethyl)amino]-3-oxetanyl}methyl)-1,2-oxazol-5-yl]methanol	1.29	Guanosine	1.25	D-(+)-Glucose	1.27
Guvacoline	1.34	Tetranor-12(S)-HETE	1.28	D-Alanyl-D-alanine	1.23	Imidazolelactic acid	1.27
2-Hydroxycinnamic acid	1.33	Acadesine	1.28	trans-10-Heptadecenoic acid	1.22	Guanine	1.27
L-Tyrosine	1.33	Benzylpiperazine	1.27	-		Hispidulin	1.26
L-Norleucine	1.32	Acetophenone	1.27	-		Sucrose	1.26
6-Aminocaproic acid	1.31	3-(2-Hydroxyethyl)indole	1.27	-		Corchorifatty acid F	1.25
Edaravone	1.30	Tyramine	1.26	-		Pyridoxine	1.24

D-(+)-Maltose	1.30	Serotonin	1.25	-		Capryloylglycine	1.24
3-(2-Hydroxy-ethyl)indole	1.25	6-Methylquino-line	1.25	-		4-Indolecarbalde-hyde	1.23
Naphtha-leneacetamide	1.23	3-Hydroxy-2-methylpyridine	1.25	-		Cyclic ADP-ribose	1.22
Imidazolelactic acid	1.22	trans-2-Ami-nomethyl-1-cy-clohexanol	1.23	-		2-Anisic acid	1.22
Flavin mononu-cleotide (FMN)	1.21	7-Methylguano-sine	1.23	-		D-(+)-Galactose	1.21
Guanine	1.21	Bethanechol	1.22	-		DL-4-Hydroxy-phenyllactic acid	1.20
-		2-Amino-4-methylpyrimi-dine	1.22	-		-	
-		D-(+)-Maltose	1.21	-		-	
-		Nicotinamide	1.21	-		-	
-		D-Raffinose	1.21	-		-	
-		Nicotinamide ad-enine dinucleo-tide (NAD+)	1.20	-		-	
